# Infection and Co-infection with Helminths and *Plasmodium* among School Children in Côte d’Ivoire: Results from a National Cross-Sectional Survey

**DOI:** 10.1371/journal.pntd.0002913

**Published:** 2014-06-05

**Authors:** Richard B. Yapi, Eveline Hürlimann, Clarisse A. Houngbedji, Prisca B. Ndri, Kigbafori D. Silué, Gotianwa Soro, Ferdinand N. Kouamé, Penelope Vounatsou, Thomas Fürst, Eliézer K. N’Goran, Jürg Utzinger, Giovanna Raso

**Affiliations:** 1 Unité de Formation et de Recherche Biosciences, Université Félix Houphouët-Boigny, Abidjan, Côte d’Ivoire; 2 Département Environnement et Santé, Centre Suisse de Recherches Scientifiques en Côte d’Ivoire, Abidjan, Côte d’Ivoire; 3 Department of Epidemiology and Public Health, Swiss Tropical and Public Health Institute, Basel, Switzerland; 4 University of Basel, Basel, Switzerland; 5 Unité de Formation et de Recherche Sciences de la Nature, Université Nangui Abrogoua, Abidjan, Côte d’Ivoire; 6 Programme National de Santé Scolaire et Universitaire, Abidjan, Côte d’Ivoire; 7 Centre for Health Policy, Imperial College London, London, United Kingdom; 8 Department of Infectious Disease Epidemiology, Imperial College London, London, United Kingdom; Liverpool School of Tropical Medicine, United Kingdom

## Abstract

**Background:**

Helminth infection and malaria remain major causes of ill-health in the tropics and subtropics. There are several shared risk factors (e.g., poverty), and hence, helminth infection and malaria overlap geographically and temporally. However, the extent and consequences of helminth-*Plasmodium* co-infection at different spatial scales are poorly understood.

**Methodology:**

This study was conducted in 92 schools across Côte d’Ivoire during the dry season, from November 2011 to February 2012. School children provided blood samples for detection of *Plasmodium* infection, stool samples for diagnosis of soil-transmitted helminth (STH) and *Schistosoma mansoni* infections, and urine samples for appraisal of *Schistosoma haematobium* infection. A questionnaire was administered to obtain demographic, socioeconomic, and behavioral data. Multinomial regression models were utilized to determine risk factors for STH-*Plasmodium* and *Schistosoma-Plasmodium* co-infection.

**Principal Findings:**

Complete parasitological and questionnaire data were available for 5,104 children aged 5-16 years. 26.2% of the children were infected with any helminth species, whilst the prevalence of *Plasmodium* infection was 63.3%. STH-*Plasmodium* co-infection was detected in 13.5% and *Schistosoma-Plasmodium* in 5.6% of the children. Multinomial regression analysis revealed that boys, children aged 10 years and above, and activities involving close contact to water were significantly and positively associated with STH-*Plasmodium* co-infection. Boys, wells as source of drinking water, and water contact were significantly and positively associated with *Schistosoma-Plasmodium* co-infection. Access to latrines, deworming, higher socioeconomic status, and living in urban settings were negatively associated with STH-*Plasmodium* co-infection; whilst use of deworming drugs and access to modern latrines were negatively associated with *Schistosoma-Plasmodium* co-infection.

**Conclusions/Significance:**

More than 60% of the school children surveyed were infected with *Plasmodium* across Côte d’Ivoire, and about one out of six had a helminth-*Plasmodium* co-infection. Our findings provide a rationale to combine control interventions that simultaneously aim at helminthiases and malaria.

## Author Summary

Parasitic worm infection and malaria are common in tropical and subtropical countries and many people suffer from multiple parasite infections concurrently. It is important to gain knowledge on co-endemicity patterns because such information can help to design and prioritize interventions and control strategies focusing on areas at highest risk of co-infection. We carried out the first national survey on parasitic worm and *Plasmodium* infection among children in 92 schools all over Côte d’Ivoire. Questionnaires were administered to determine the children's socioeconomic status and infection-related risk factors. We found an overall prevalence of *Plasmodium* infection of 63.3%. Hookworm was the most common parasitic worm infection (17.2%), whereas schistosome worms were more focally distributed (9.1%). One out of six children was co-infected with parasitic worms and *Plasmodium*. Children from the poorest households living in rural areas were at highest risk of co-infection. The lack of sanitation facilities, and activities like swimming or fishing in nearby rivers were found to be positively associated with multiple species parasite infection. Our study motivates the integration of combined intervention measures that concurrently target neglected tropical diseases and malaria in highly resource-constrained settings.

## Introduction

Malaria is still threatening the life of millions of people in sub-Saharan Africa, although control efforts have decreased mortality attributable to this disease by 30% between 2004 and 2010 [1]. Malaria often overlaps with parasitic worm infections, such as soil-transmitted helminths (STHs) and schistosomes. Along with HIV/AIDS, tuberculosis, and anemia, malaria and helminthiases are the main causes of years lived with disability (YLDs) in sub-Saharan Africa [2], and negatively affect the social and economic development [3], [4].

Current malaria control tools and strategies consist of prompt diagnosis and treatment with artemisinin-based combination therapy (ACT), use of long-lasting insecticidal nets (LLINs), indoor residual spraying (IRS), and strengthening of health systems, among others [5]. For the control of helminthiases, preventive chemotherapy is the current global strategy of choice [6]. Whenever resources allow, other interventions, including information, education, and communication (IEC), provision of improved sanitation, and clean water are being advocated [7]–[9]. Combining control efforts targeting multiple diseases (e.g., malaria and helminthiases), might be more cost-effective than targeting single diseases, given that co-infections might have additive or even multiplicative effects on clinical outcomes such as anemia [10].

Helminth and *Plasmodium* infections are likely to proliferate under favorable climatic and environmental conditions, particularly among the poorest communities that lack essential facilities, such as clean water and sanitation [9], [11]. Whilst malaria is governed by climatic factors linked to vector ecology and parasite development [12], schistosomiasis is intimately connected with human-water contacts and the presence of infected intermediate host snails [13]. In Tanzania, for example, the risk of malaria-schistosomiasis co-infection has been shown to be higher for children living in agro-ecosystems that are characterized by irrigated rice farming [14]. In Cameroon, *Plasmodium*-helminth co-infection was associated with altitude [15]. It is important to understand how exactly climatic, environmental, and socioeconomic factors influence the distribution of single and multiple species parasite infections, since this can help to better target control approaches at different spatial scales.

In Côte d’Ivoire, helminth infection and malaria remain important public health issues. Considerable efforts are underway to enhance control interventions after the socio-political crisis that occurred between 2002 and 2011, which deteriorated the health and education systems [16], [17]. For example, LLINs were distributed to the most vulnerable population groups, free of charge, whilst ACT and rapid diagnostic tests (RDTs) were made available by the national malaria control program and non-governmental organizations. Furthermore, anthelmintic treatment was provided to preschoolers and school-aged children in conjunction with national vaccination campaigns and school-based treatment campaigns.

The aim of this study was to deepen the understanding of helminth-*Plasmodium* co-infection at the national level in Côte d’Ivoire. Hence, a country-wide cross-sectional survey was carried out during the dry season from November 2011 to February 2012. Results are discussed in the context of spatial targeting of control interventions that are aimed at multiple parasite infection simultaneously.

## Methods

### Ethics Statement

This study received clearance from the ethics committees of Basel (EKBB; reference no. 30/11), and Côte d’Ivoire (reference no. 09-2011/MSHP/CNER-P). Additionally, permission to carry out the study was sought from the Ministry of National Education. Before visiting any school, the district health and education authorities, school directors, and teachers were informed about the aim and procedures of the study. Written informed consent was obtained from the parents/guardians of children, whereas children assented orally. Participation was voluntary, and hence, withdrawal was possible anytime without further obligation. Parasitological and questionnaire data were coded and treated confidentially.

All children were treated free of charge with a single oral dose of albendazole (400 mg). Children with a positive RDT and clinical signs of malaria were given ACT, adhering to standard procedures in Côte d’Ivoire. Children with *Schistosoma* infection were treated with a single oral dose of praziquantel (40 mg/kg). In schools where the observed *Schistosoma* point prevalence exceeded 25%, all children were given praziquantel.

### Study Area and Design

**Figure 1:**
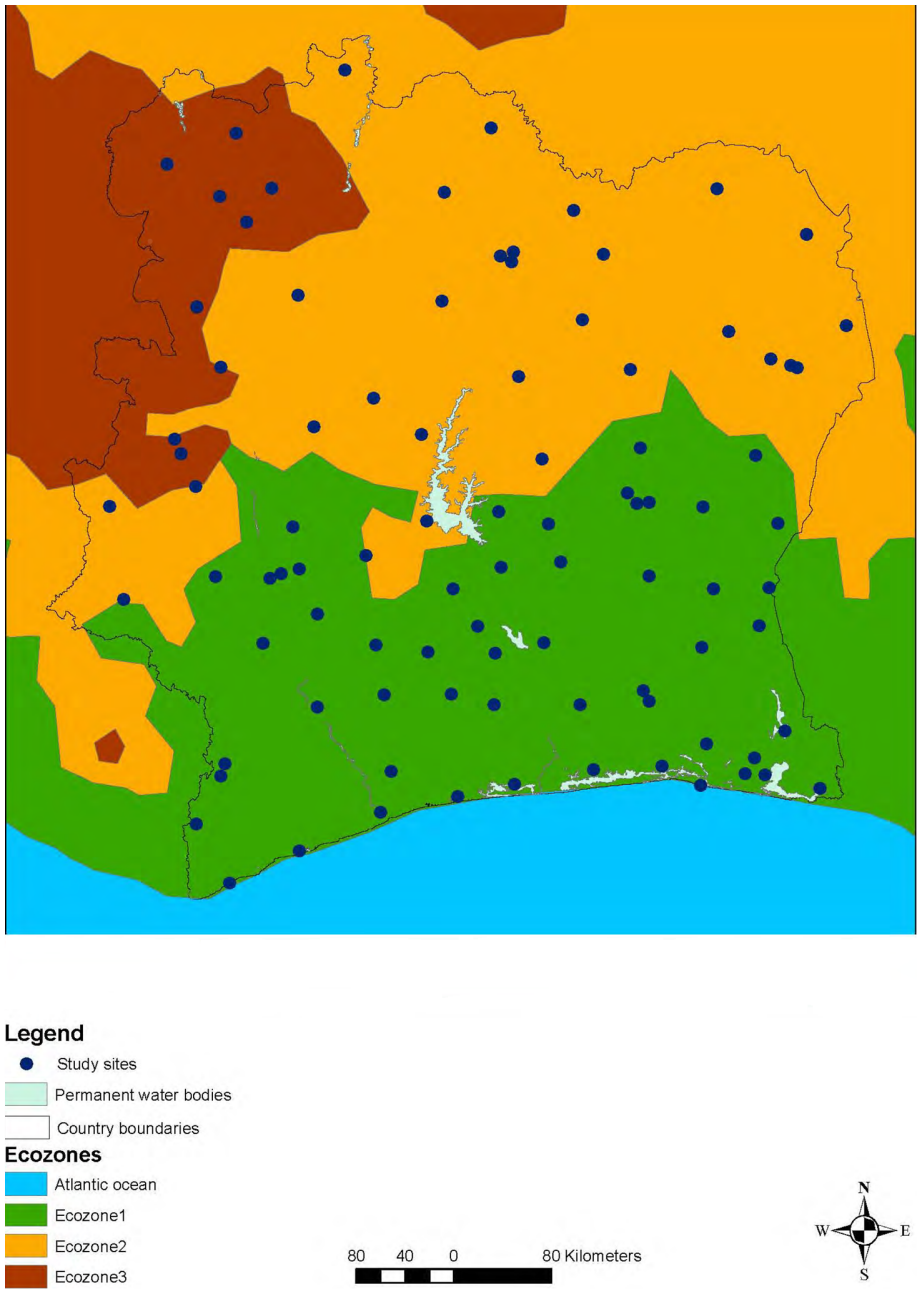
Surveyed schools (N = 92) in a national cross-sectional survey carried out in Côte d’Ivoire between November 2011 and February 2012.

Côte d’Ivoire is located in West Africa, covers an area of 322,462 km^2^, and is situated between latitudes 4^o^30′ and 10^o^30′ North, and longitudes 2^o^30′ and 8^o^30′ West. In 2011, the population of Côte d’Ivoire was estimated at 21 million. Data from satellite imagery (i.e., rainfall, elevation, normalized difference vegetation index, land cover, and maximum land surface temperature) were utilized to delineate different ecozones. In brief, the data were imported in ERDAS Imagine 9.3 software and an iterative self-organizing data analysis technique (ISODATA) was used on the environment factors to create different ecozones based on between-class similarities [18]. The three resulting ecozones were: (i) the South and western parts with abundant rainfall and forest as dominant vegetation, and with mountainous landscape in the West; (ii) the northern part with less rainfall and savannah as vegetation; and (iii) the northwestern part characterized by savannah but more rainfall than the North (Figure 1).

We designed a country-wide cross-sectional survey using the lattice plus close pairs design [19]. Our aim was to select 100 schools across the country. It was determined whether there was a primary school comprising a minimum of 60 children attending grades 3 to 5. Ninety-four of the 100 selected schools met this criterion, hence were eligible to participate. The sample size per school was delimited to 60 children, which exceeds the minimum sample size of 50, as recommended by the World Health Organization (WHO) for collection of baseline information pertaining to helminth prevalence and intensity in the school-aged population within large-scale surveys [20].

### Study Procedures

District education officers were informed about the aims and procedures of the study and which schools had been selected in their district. School directors and teachers were contacted and invited to inform parents or legal guardians of 60 children attending grades 3 and 4. If there were less than 60 children in grades 3 and 4, additional children were selected from grade 5 to reach the intended sample size of 60 children per school.

The survey team visited one school per day. Geographical coordinates of schools were recorded using a hand-held global positioning system (GPS) receiver (Garmin Sery GPS MAP 62; Olathe, United States of America). Children were informed about the objectives and procedures of the study. Teachers were invited to prepare class list of those children whose parents/guardians had provided written informed consent. The lists contained the children's name, age, sex, and school grade. All children were subjected to parasitological examination and a questionnaire. Unique identification numbers were assigned to participating children.

Two plastic containers (25 ml and 10 ml) were distributed to all participants, labeled with unique identification numbers. Children were asked to return the first container with a portion of their fresh morning stool and the second container with a urine sample. Additionally, a finger prick blood sample was taken for *Plasmodium falciparum* examination, using an RDT (ICT ML01 malaria Pf kit; ICT Diagnostics, Cape Town, South Africa). Thick and thin blood films were prepared on microscope slides, air-dried, and transferred to nearby laboratories.

### Laboratory Procedures

Stool, urine and blood samples were processed on the day of collection. In brief, for each stool sample, duplicate Kato-Katz thick smears, using 41.7 mg plastic templates, were prepared. After a clearing time of 30–45 min, the Kato-Katz thick smears were examined under a microscope, and the number of eggs of *Schistosoma mansoni*, hookworm, *Ascaris lumbricoides*, *Trichuris trichiura*, and any other helminths were counted and recorded for each species separately. Urine samples were screened for microhematuria, using reagent strips (Hemastix; Siemens Healthcare Diagnostics GmbH, Eschborn, Germany) as a proxy for *S. haematobium* infection [21]. Reagent strips were dipped into the urine sample and, after 1 min, the color change was recorded following the manufacturer's instruction. The presence of blood in urine was denoted by a change in color from yellow to yellow mixed with green spots, green, or darkish green. Blood films were stained with 10% Giemsa and *Plasmodium* species identified under a microscope. Parasitemia was determined by experienced laboratory technicians, counting the number of *Plasmodium* parasites per 200 white blood cells (WBC). If less than 10 parasites were recorded, the reading was continued up to 500 WBC. Counts were converted to number of parasites per μl of blood, assuming a WBC count of 8,000 per μl of blood.

For quality control, 10% of the Kato-Katz thick smears and Giemsa-stained blood films were randomly selected and re-examined by a senior laboratory technician. In case of discordant results (e.g., negative *vs.* positive readings; parasitemia or helminth egg counts differing by more than 10%), slides were reexamined by a third technician and results discussed until agreement was reached.

### Questionnaire Survey

A questionnaire was administered in order to derive a household wealth index, and to identify potential risk factors of parasite infections, as done previously [22]. In brief, for appraisal of household's socioeconomic status, we employed an asset-based approach on ownership (i.e., bicycle, car, cooker, DVD/VCD, fridge, motorbike, phone/mobile phone, radio, TV set, and fan) and housing characteristics (access to the power grid and wall type). Presence of latrine was noted, distinguishing between modern latrines (made of cement and possibly flush) and traditional latrines (made with local materials, such as clay and wood). Furthermore, questions pertaining to typical habits, such as wearing shoes, washing hands after defecation, drinking water source, sleeping under a bed net, use of insecticides, and water-contact patterns (i.e., swimming or fishing) were included in the questionnaire. Children were also asked whether they had received anthelmintic treatment or antimalarials (traditional or modern treatment) in the past 2 weeks. Moreover, the questionnaire included eight diseases and eight symptoms and was pre-tested in a school not involved in the current survey. Teachers were trained and they administered the questionnaire together with the team.

### Statistical Analysis

Data were double-entered and cross-checked in EpiInfo version 3.5.3 (Center for Diseases Control and Prevention; Atlanta, United States of America) and analyzed in STATA version 12 (STATA Corp.; College Station, United States of America). Only children who had complete datasets (i.e., stool examination with duplicate Kato-Katz thick smears, urine examination using reagent strips, finger prick blood samples subjected to RDT and thick and thin blood films, and complete questionnaire data) were considered for further analysis.

Geographical data were displayed in ArcView GIS version 9.0 (Environmental Systems Research Institute Inc.; Redlands, United States of America). Children were subdivided into two age groups (5–9 and 10–16 years). For helminths, an infection was defined as the presence of at least one parasite egg on at least one of the two Kato-Katz thick smears. Helminth infection intensities were determined by multiplying the sum of the two Kato-Katz readings with a factor of 12 and expressed as eggs per 1 g of stool (EPG). Infection intensities were stratified into light, moderate, and heavy, according to WHO guidelines [23] The cut-offs to distinguish between light and moderate and between moderate and heavy infections were 100 and 400 EPG for *S. mansoni*, 2,000 and 4,000 for hookworm; 5,000 and 50,000 for *A. lumbricoides*, and 1,000 and 10,000 for *T. trichiura*. Microhematuria was used as proxy for *S. haematobium* infection. As a positive test, we considered those reagent strips that showed a distinct color change from yellow to green according to the manufacturer's instructions. Trace results were considered as *S. haematobium*-negative. *Plasmodium* spp. infection was specified on the basis of RDT and blood film examination. A child was considered positive for *P. falciparum* when blood films and the RDT were positive. *P. ovale* and *P. malaria* were assessed only through thick and thin blood films. Infection intensity was stratified into four categories, namely (i) 1–50; (ii) 51–500; (iii) 501–5,000; and (iv) >5,000 parasites/μl of blood [24].

The socioeconomic status of a child was determined using the aforementioned asset-based approach at the household level. Asset weights were obtained using principal component analysis (PCA) [25]. For the covariate ecozone, we only considered two categories, the area of the third ecozone being very small (only seven schools were located there). The two ecozones considered were (i) the savannah-like ecozone in the North and (ii) the forest-like ecozone in the South.

The prevalence of single species parasite infection and intensity (i.e., specific infection regardless of other infections) for helminths and *Plasmodium* were tested for associations with sex and age group, using logistic and negative binomial regression models, respectively. We accounted for cluster effects at the unit of the school. Associations between parasite species were assessed using multivariate logistic regression models on a single parasite species/parasite group with all other parasite species as covariates. Cluster effects were introduced into these models and a stepwise backward elimination process was adopted to include associated covariates at a significance level of 0.2. Based on epidemiological and control program considerations, we grouped investigated parasite species into STHs (i.e., hookworm, *A. lumbricoides*, and *T. trichiura)*, *Schistosoma* (i.e., *S. mansoni* and microhematuria), and *Plasmodium* spp. for further STH*-Plasmodium* and *Schistosoma*-*Plasmodium* co-infection analysis. For this purpose, two new outcome variables were created with four categories; (i) co-infection with STHs or *Schistosoma* and *Plasmodium*; (ii) mono-infection with STHs or *Schistosoma*; (iii) mono-infection with *Plasmodium*; and (iv) no infection. To analyze co-infection patterns of STH-*Plasmodium* or *Schistosoma*-*Plasmodium*, we performed multinomial regressions that accounted for cluster effect at the unit of the school. We used a stepwise backward elimination process to identify risk factors associated with mono- and co-infections. Risk factors were accepted at a significance level of 0.2.

## Results

### Study Participation

**Figure 2:**
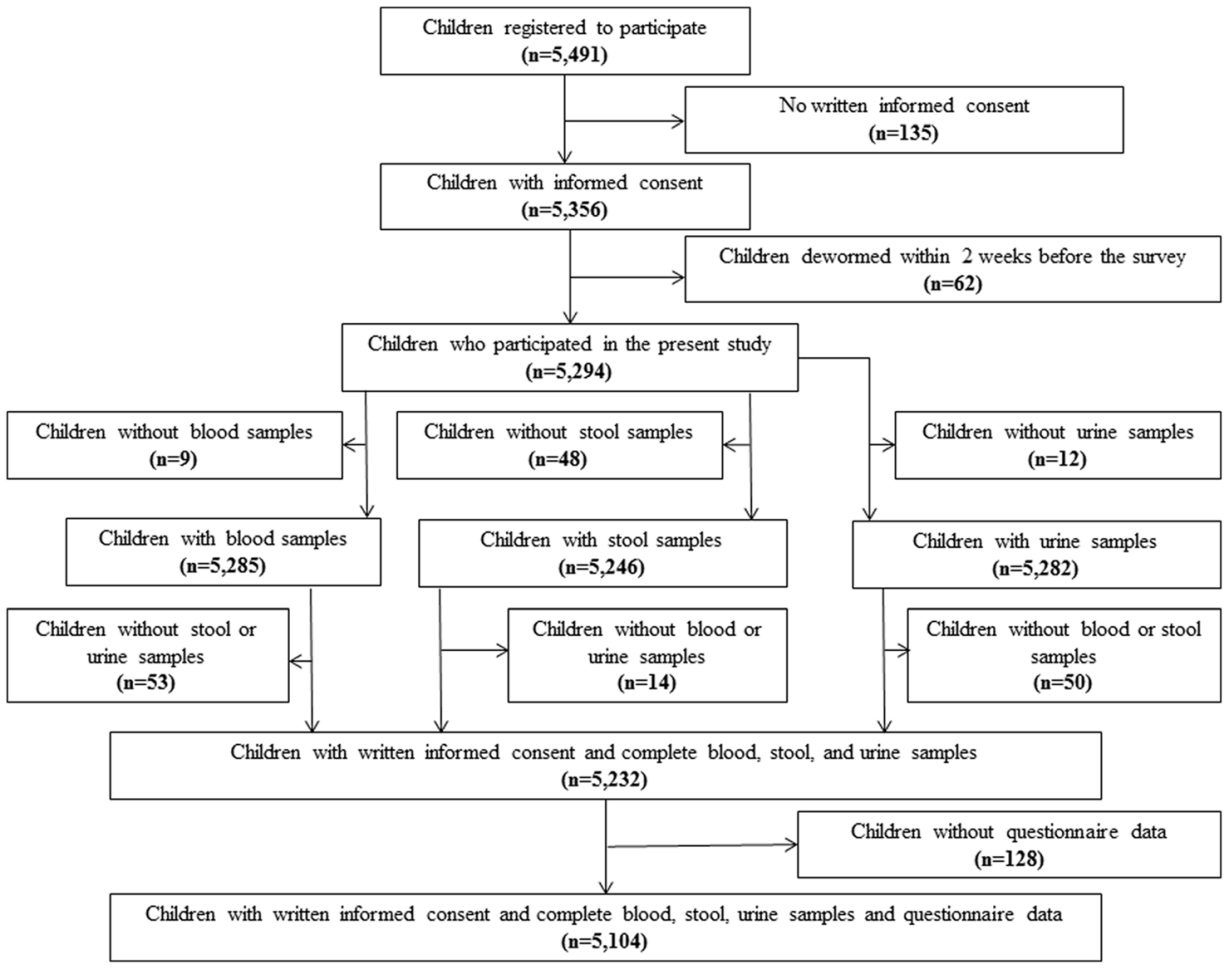
Flow chart showing study compliance from a national cross-sectional survey carried out in 92 schools in Côte d’Ivoire between November 2011 and February 2012.

Figure 1 shows the survey locations across Côte d’Ivoire. From the 94 schools randomly selected, one refused to participate. Overall, 5,491 children were invited to participate, 4,252 (77.4%) of whom lived in rural areas and the remaining 1,239 (22.6%) in urban settings. The majority of children (n = 5,356, 97.5%) had written informed consent from their parents/guardians. Children from one school were not included in the analysis (n = 62), as they underwent mass deworming 2 weeks before our team passed. A total of 5,232 (95.3%) children had complete parasitological data. Among them, 128 children lacked questionnaire data. Hence, 5,104 (93.0%) children (2,391 girls and 2,713 boys; 2,226 aged 5–9 years, and 2,878 aged 10–16 years) had complete datasets (Figure 2).

### Helminth and *Plasmodium* Infections

Single parasite infection prevalence data and associations with sex and age groups are summarized in Table 1. Among the 5,246 children who provided stool samples, the prevalence of helminth infections were as follows: hookworm, 17.2% (range 0–60.0% at the unit of the school); *A. lumbricoides*, 1.9% (range: 0–37.0%), *T. trichiura* 1.3% (range: 0–22.2%); and *S. mansoni* 3.7% (range: 0–41.1%). With regard to *S. mansoni* infection, we found highest prevalences in the western part of the country. Among the 5,282 children who provided urine samples, microhematuria was detected in 5.7% of the children (range: 0–79.6%) with the highest rates in the south and south-eastern part of the country.

**Table 1:** Parasite infection prevalences and associations with sex and age group.

Parasite	Covariate	Covariate category	N	Positive (%)	OR (95% CI)	P-value
Hookworm			5,246	903 (17.2)		
	Sex	Girls	2,444	271 (11.1)	1.0	
		Boys	2,802	632 (22.6)	**2.3 (1.9, 2.9)**	**<0.001**
	Age group	5–9 years	2,304	261 (11.3)	1.0	
		10–16 years	2,942	642 (21.8)	**2.2 (1.8, 2.7)**	**<0.001**
*A. lumbricoides*			5,246	97 (1.9)		
	Sex	Girls	2,444	44 (1.8)	1.0	
		Boys	2,802	53 (1.9)	1.1 (0.7, 1.6)	0.823
	Age group	5–9 years	2,304	32 (1.4)	1.0	
		10–16 years	2,942	65 (2.2)	**1.6 (1.1, 2.3)**	**0.013**
*T. trichiura*			5,246	66 (1.3)		
	Sex	Girls	2,444	28 (1.2)	1.0	
		Boys	2,802	38 (1.4)	1.2 (0.7, 1.9)	0.492
	Age group	5–9 years	2,304	21 (0.9)	1.0	
		10–16 years	2,942	45 (1.5)	1.7 (0.9, 3.0)	0.080
*S. mansoni*			5,246	193 (3.7)		
	Sex	Girls	2,444	47 (1.9)	1.0	
		Boys	2,802	146 (5.2)	**2.8 (1.8, 4.5)**	**<0.001**
	Age group	5–9 years	2,304	53 (2.3)	1.0	
		10–16 years	2,942	140 (4.8)	**2.1 (1.5, 2.9)**	**<0.001**
*S. haematobium*			5282	299 (5.7)		
	Sex	Girls	2,460	137 (5.6)	1.0	
		Boys	2,822	162 (5.8)	1.0 (0.8, 1.3)	0.793
	Age group	5–9 years	2,327	115 (4.9)	1.0	
		10–16 years	2,955	184 (6.2)	1.3 (0.9, 1.8)	0.161
*P. falciparum*			5,285	3,270 (61.9)		
	Sex	Girls	2,461	1,439 (58.5)	1.0	
		Boys	2,824	1,831 (64.8)	**1.3 (1.2, 1.5)**	**<0.001**
	Age group	5–9 years	2,327	1,433 (61.6)	1.0	
		10–16 years	2,958	1,837 (62.1)	1.0 (0.9, 1.2)	0.771
*P. malariae*			5,285	198 (3.7)		
	Sex	Girls	2,461	88 (3.6)	1.0	
		Boys	2,824	110 (3.9)	1.1 (0.8, 1.5)	0.557
	Age group	5–9 years	2,327	99 (4.3)	1.0	
		10–16 years	2,958	99 (3.3)	0.8 (0.6, 1.1)	0.121
*P. ovale*			5,285	16 (0.3)		
	Sex	Girls	2,461	7 (0.3)	1.0	
		Boys	2,824	9 (0.3)	1.1 (0.5, 2.5)	0.782
	Age group	5–9 years	2,327	8 (0.3)	1.0	
		10–16 years	2,958	8 (0.3)	0.8 (0.4, 1.7)	0.554

Parasite infection prevalences stem from a national survey conducted in Côte d’Ivoire between November 2011 and February 2012. Children from 92 different schools were parasitologically tested. The prevalences are given in percentages. Associations were assessed using bivariate logistic regression models that account for cluster effects at the unit of the school. Statistically significant (p<0.05) odds ratios are highlighted in bold.

N = number of children.

OR = odds ratio.

CI = confidence interval.

**Figure 3:**
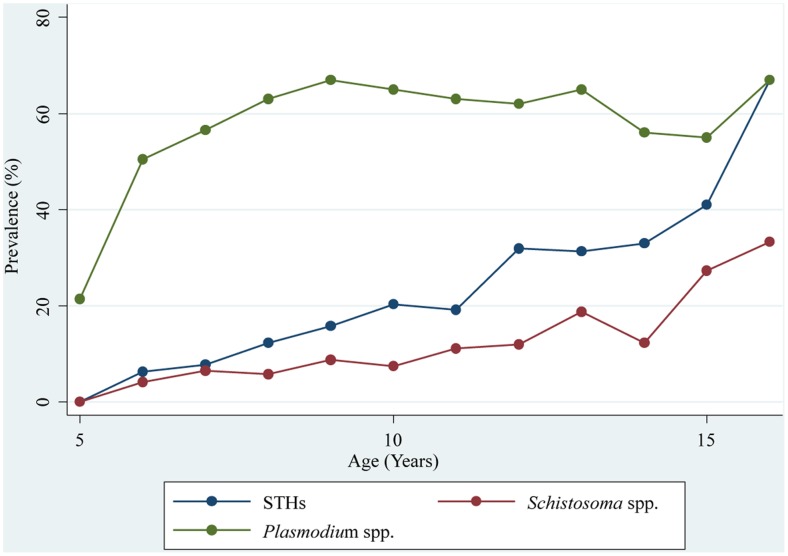
Age-prevalence curves for soil-transmitted helminths (STH), *Schistosoma* spp., and *Plasmodium* spp. Data were obtained from a national cross-sectional survey in 92 schools in Côte d’Ivoire between November 2011 and February 2012.

Boys had a significantly higher infection prevalence with hookworm compared to girls (22.6% *vs*. 11.1%, odds ratio (OR) = 2.3; p<0.001). Children aged 10 years and above showed a considerably higher hookworm infection prevalence than their younger counterparts (21.8% *vs*. 11.3%, OR = 2.2; p<0.001). For *A. lumbricoides*, we found that older children were significantly more infected compared to the younger age group (2.2% *vs*. 1.4%; OR = 1.6; p = 0.013). For *T. trichiura*, there were no statistically significant differences between sex and age groups. Boys were more often infected with *S. mansoni* than girls (5.2% *vs*. 1.9%; OR = 2.8; p<0.001) and older children had a 2-fold higher prevalence than their younger counterparts (4.8% *vs*. 2.3%; OR = 2.1; p<0.001). For *S. haematobium*, proxied by the presence of microhematuria, no statistically significant difference was found between sex and the two age groups. Age-prevalence curves of STHs and *Schistosoma* are displayed in Figure 3.

*Plasmodium* spp. was the most frequent infection with an overall prevalence of 63.3%. *P. falciparum* was the predominant species (61.9%). At the unit of the school, the prevalence ranged from 13.1% to 88.3%. Boys showed a somewhat higher infection prevalence than girls (64.8% *vs*. 58.5%; OR = 1.3; p<0.001). No statistically significant difference was found among the two age groups (61.6% *vs*. 62.1%; OR = 1.0; p = 0.897). The age-prevalence curve is shown in Figure 3. *P. malariae* and *P. ovale* showed low prevalences; 3.7% (range at the unit of school: 0–15.0%) and 0.3% (range: 0–3.3%), respectively. No statistically significant differences were found between sex and age group for these two *Plasmodium* species. 2.7% of the children were tested positive for mixed *Plasmodium* species infection.

### Helminth Infection Intensities and *Plasmodium* Parasitemia

Most helminth infections were of light intensity (Table 2). In fact, 98.5%, 96.8%, 85.6%, and 49.7% of *T. trichiura*, hookworm, *A. lumbricoides*, and *S. mansoni* infections, respectively, were classified as light. There was no statistically significant difference in helminth infection intensities between sex and age groups for any of the helminth species encountered, as assessed by negative binomial regression models on fecal egg counts.

**Table 2:** Parasite mean infection intensities and infection intensity categories and association of infection intensity with sex and age group.

Parasites	Covariate	Covariate category	Mean infection intensity (EPG)	Infection intensity categories N (%)				IRR (95% CI)	P-value
				Light	Moderate	Heavy			
Hookworm			388	874 (96.8)	18 (2.0)	11 (1.2)			
	Sex	Girls	371	264 (97.4)	3 (1.1)	4 (1.5)		1.0	
		Boys	395	610 (96.5)	15 (2.4)	7 (1.1)		1.1 (0.7, 1.6)	0.777
	Age group	5–9 years	365	253 (97.0)	4 (1.5)	4 (1.5)		1.0	
		10–16 years	398	621 (96.6)	14 (2.2)	7 (1.1)		1.1 (0.8, 1.6)	0.639
*A. lumbricoides*			2,060	83 (85.6)	14 (14.4)	0 (0.0)			
	Sex	Girls	1,815	38 (86.4)	6 (13.6)	0 (0.0)		1.0	
		Boys	2,264	45 (84.9)	8 (15.1)	0 (0.0)		1.2 (0.8, 1.8)	0.264
	Age group	5–9 years	1,787	28 (87.5)	4 (12.5)	0 (0.0)		1.0	
		10–16 years	2,195	55 (84.6)	10 (15.4)	0 (0.0)		1.2 (0.7, 2.0)	0.425
*T. trichiura*			174	65 (98.5)	1 (1.5)	0 (0.0)			
	Sex	Girls	114	28 (100.0)	0 (0.0)	0 (0.0)		1.0	
		Boys	219	37 (97.4)	1 (2.6)	0 (0.0)		1.9 (0.8, 4.9)	0.165
	Age group	5–9 years	274	20 (95.2)	1 (4.8)	0 (0.0)		1.0	
		10–16 years	127	45 (100.0)	0 (0.0)	0 (0.0)		0.5 (0.1, 1.7)	0.251
*S. mansoni*			273	96 (49.7)	65 (33.7)	32 (16.6)			
	Sex	Girls	285	24 (51.0)	13 (27.7)	10 (21.3)		1.0	
		Boys	269	72 (49.3)	52 (35.6)	22 (15.1)		0.9 (0.6, 1.5)	0.796
	Age group	5–9 years	302	31 (58.5)	14 (26.4)	8 (15.1)		1.0	
		10–16 years	262	65 (46.4)	51 (36.4)	24 (17.1)		0.9 (0.5, 1.6)	0.641

Parasites	Covariate	Covariate category	Mean infection intensity (parasites/μl of blood)	Infection intensity categories N (%)					IRR (95% CI)		P-value	
				1–50	51–500	501–5000	>5000					
*P. falciparum*			1,613	135 (4.1)	1,317 (40.4)	1,601 (49.1)	210 (6.4)					
	Sex	Girls	1,684	58 (4.0)	601 (41.8)	678 (47.2)	100 (7.0)		1.0			
		Boys	1,557	77 (4.2)	716 (39.2)	923 (50.6)	110 (6.0)		0.9 (0.8, 1.1)		0.288	
	Age group	5–9 years	1,950	42 (2.9)	497 (34.8)	777 (54.3)	114 (8.0)		1.0			
		10–16 years	1,350	93 (5.1)	820 (44.7)	824 (45.0)	96 (5.2)		**0.7 (0.6, 0.8)**		**<0.001**	
*P. malariae*			1,196	5 (2.5)	106 (53.5)	78 (39.4)	9 (4.6)					
	Sex	Girls	1,374	3 (3.4)	48 (54.5)	32(36.4)	5 (5.7)		1.0			
		Boys	1,054	2 (1.8)	58 (52.7)	46 (41.8)	4 (3.6)		0.8 (0.4, 1.4)		0.369	
	Age group	5–9 years	1,494	0 (0.0)	47 (47.5)	46 (46.5)	6 (6.1)	1.0				
		10–16 years	898	5 (5.1)	59 (59.6)	32 (32.3)	3 (3.0)		**0.6 (0.4, 1.0)**		**0.041**	
*P. ovale*			793	0 (0.0)	9 (60.0)	6 (40.0)	0 (0.0)					
	Sex	Girls	1,003	0 (0.0)	4 (66.7)	2 (33.3)	0 (0.0)		1.0			
		Boys	653	0 (0.0)	5 (55.6)	4 (44.4)	0 (0.0)		0.7 (0.1, 3.3)		0.607	
	Age group	5–9 years	976	0 (0.0)	4 (50.0)	4 (50.0)	0 (0.0)		1.0			
		10–16 years	584	0 (0.0)	5 (71.4)	2 (28.6)	0 (0.0)		0.6 (0.1, 3.0)		0.534	

Parasite infection intensities stem from a national survey conducted in Côte d'Ivoire between November 2011 and February 2012. Children from 92 different survey locations were parasitologically tested. Infection intensity defined as the number of eggs per 1 g of stool (EPG) in the case of helminths infections and the number of parasites per μl of blood in the case of *Plasmodium.* Helminth infection intensity categories were defined according to World Health Organization guidelines [23] whereas cut-offs for *Plasmodium* parasitemia were applied according to previous studies conducted in the country [24]; both are reported as percentage of all positive cases of the respective parasite infection. Associations were assessed by negative binomial regression models on parasite counts, accounting for cluster effects at school unit. Statistically significant (p<0.05) IRR are highlighted in bold.

N = number of children.

IRR =  incidence rate ratio.

CI = confidence interval.

The mean *P. falciparum* parasitemia among infected children was 1,613 parasites/μl of blood (Table 2). Most of the *P. falciparum* infections showed a parasitemia not exceeding more than 5000 parasites/μl of blood (93.6%). There was no difference in parasitemia between males and females (incidence risk ratio (IRR) = 0.9; p = 0.288). Older children had a somewhat lower IRR of parasitemia than their younger counterparts (1,350 *vs*. 1,950 parasites/μl of blood; IRR = 0.7; p<0.001). A similar pattern was found for *P. malariae*, which showed a mean parasitemia of 1,196 parasites/μl of blood, a majority of the infections ranging in the intensity categories of 51–500 (53.5%) and 501–5000 (39.4%) parasites/μl of blood, and a decreased IRR in older children compared to their younger peers (898 *vs.* 1,494 parasites/μl of blood; IRR = 0.6; p = 0.041). No statistical difference with regard to *P. ovale* parasitemia was found between sex and age groups.

### Parasite-Parasite Associations

Results from parasite-parasite association inferred from multivariate logistic regression models that account for cluster effect at the unit of the school are displayed in the supplementary Table S1. In brief, several significant positive associations were found between parasites. For example, STH infection was positively associated with *P. falciparum* infection. Hookworm infection was positively associated with *T. trichiura*, *S. mansoni* and *P. falciparum*. In the *A. lumbricoides* model, *T. trichiura* was the only significantly and positively associated parasite, whereas in the *T. trichiura* model, *A. lumbricoides* and hookworm were found to be positively associated. In the *S. mansoni*, *Plasmodium* spp., and *P. falciparum* infection models, hookworm infection was positively associated.

### Helminth-*Plasmodium* Co-infection and Risk Profiles

Results from the STH-*Plasmodium* co-infection model are displayed in Table 3. A total of 689 children were co-infected with STHs and *Plasmodium* spp., 286 were infected with STHs only, and 2,543 with *Plasmodium* spp. only. Wealth, living in urban settings, presence of any type of latrine, and access to deworming drugs were negatively associated with STH-*Plasmodium* co-infection. In contrast, boys, children aged 10–16 years, and children reporting to use river as source of drinking water were at higher risk of STH-*Plasmodium* co-infection. For mono-infections with STHs, significant negatively associated variables included wealth, urban setting, savannah-like ecozone and traditional latrines, while male sex and older age were positively associated variables. *Plasmodium* mono-infection had wealth, urban setting, presence of modern latrine, and access to deworming drugs as negatively associated factors. Male sex, the savannah-like ecozone, and use of well water as source of drinking water were positively associated with *Plasmodium* mono-infections.

**Table 3:** Associated risk factors for STHs and *Plasmodium* mono- and co-infection among 5,104 school children.

Covariates	Covariate category	STH mono infection			*Plasmodium* mono infection			STH-*Plasmodium* co-infection		
		Positive (%)	RRR	P-value	Positive (%)	RRR	P-value	Positive (%)	RRR	P-value
		286 (5.6)			2,543 (49.8)			689 (13.5)		
Sex	Girls		1.0			1.0			1.0	
	Boys		**1.9 (1.4, 2.6)**	**<0.001**		**1.2 (1.1, 1.4)**	**0.001**		**2.5 (1.9, 3.2)**	**<0.001**
Age group	5–9 years		1.0			1.0			1.0	
	10–16 years		**2.2 (1.6, 3.1)**	**<0.001**		0.9 (0.8, 1.1)	0.477		**1.7 (1.4, 2.2)**	**<0.001**
Socioeconomic status	Most poor		1.0			1.0			1.0	
	Very poor		1.0 (0.7, 1.5)	0.926		1.0 (0.8, 1.3)	0.948		0.8 (0.6, 1.0)	0.093
	Poor		0.8 (0.5, 1.1)	0.185		1.0 (0.8, 1.3)	0.793		**0.5 (0.3, 0.7)**	**<0.001**
	Less poor		**0.6 (0.4, 0.9)**	**0.011**		**0.7 (0.6, 0.9)**	**0.010**		**0.3(0.2, 0.4)**	**<0.001**
	Least poor		**0.4 (0.2, 0.6)**	**<0.001**		**0.7 (0.5, 0.9)**	**0.018**		**0.2 (0.1, 0.4)**	**<0.001**
Setting	Rural		1.0			1.0			1.0	
	Urban		**0.6 (0.4, 0.9)**	**0.029**		**0.7 (0.5, 1.0)**	**0.047**		**0.5 (0.2, 0.9)**	**0.029**
Ecozone	Forest-like		1.0			1.0			1.0	
	Savannah-like		**0.7 (0.5, 1.0)**	**0.047**		**1.4 (1.1, 1.8)**	**0.003**		1.2 (0.7, 1.9)	0.497
Drinking water source	Tap/pump water		1.0			1.0			1.0	
	Well water		1.0 (0.6, 1.5)	0.905		**1.4 (1.1, 1.7)**	**0.004**		1.2 (0.8, 1.8)	0.337
	River or pond water		1.1 (0.6, 1.7)	0.856		**1.3 (1.0, 1.6)**	**0.025**		1.5 (1.0, 2.3)	0.057
Sanitation	No latrine		1.0			1.0			1.0	
	Traditional latrine		**0.6 (0.4, 0.9)**	**0.014**		0.9 (0.7, 1.1)	0.330		**0.6 (0.4, 0.9)**	**0.008**
	Modern latrine		0.6 (0.3, 1.2)	0.177		**0.6 (0.4, 0.8)**	**0.001**		**0.4 (0.2. 0.7)**	**0.001**
Water-related activities	Had no activity		1.0			1.0			1.0	
	Had activity		1.4 (0.9, 2.1)	0.097		1.1 (0.9, 1.3)	0.593		**1.4 (1.0, 1.8)**	**0.030**
Deworming	Had no treatment in the past 2 weeks		1.0			1.0			1.0	
	Had treatment in the past 2 weeks		0.8 (0.5, 1.1)	0.125		**0.8 (0.7, 0.9)**	**0.010**		**0.7 (0.5, 0.9)**	**0.016**
Malaria treatment	Had no treatment in the past 2 weeks		1.0			1.0			1.0	
	Had treatment in the past 2 weeks		1.1 (0.7, 1.6)	0.743		0.9 (0.7, 1.0)	0.119		0.8 (0.6, 1.1)	0.108

Parasite mono- and co-infection prevalences and risk factors associated stem from a national survey conducted in Côte d'Ivoire between November 2011 and February 2012. Children from 92 different survey locations were parasitologically tested and socioeconomic status assessed together with potential risk-related behavior and preventive measures taken. The prevalences are given in percentages. Associations were assessed using a multinomial regression model adjusted for *Schistosoma* spp., and accounting for cluster effects at school unit. Statistically significant (p<0.05) RRR are highlighted in bold.

STH = soil-transmitted helminths.

RRR = relative risk ratio.

CI = confidence interval.

Results from the *Schistosoma*-*Plasmodium* co-infection model are shown in Table 4. A total of 284 schoolchildren were co-infected with both parasite groups, 178 were identified with *Schistosoma* worms only and 2,948 had *Plasmodium* infections only. *Schistosoma*-*Plasmodium* co-infection was negatively associated with the use of deworming drugs and showed a negative trend for presence of a modern latrine in the household. In contrast, co-infection was positively associated with male sex, use of well water as drinking source and activities in or close to water bodies like swimming and fishing. For *Schistosoma* mono-infection, regular hand washing was negatively associated with this infection group, whilst older age group, well water as drinking water, and contact with water body (swimming and/or fishing) were positively associated. For *Plasmodium* mono-infection, higher socioeconomic status, living in an urban area, presence of a modern latrine and deworming were negatively associated with this infection group. Thus, male sex, a savannah-like ecozone, and open surface waters (including well, river, pond and swamp) as source for drinking water were associated with higher risk of a *Plasmodium* spp. mono-infection.

**Table 4:** Associated risk factors for *Plasmodium* and *Schistosoma* mono- and co-infection among 5,104 school children.

Covariates	Covariate category	*Schistosoma* mono-infection			*Plasmodium* mono-infection			*Schistosoma-Plasmodium* co-infection		
		Positive (%)	RRR	P-value	Positive (%)	RRR	P-value	Positive (%)	RRR	P-value
		178 (3.5)			2,948 (57.8)			284 (5.6)		
Sex	Girls		1.0			1.0			1.0	
	Boys		1.3 (0.9, 1.8)	0.201		**1.2 (1.1, 1.4)**	**<0.001**		**1.7 (1.3, 2.3)**	**0.002**
Age groups	5–9 years		1.0			1.0			1.0	
	10–16 years		**1.6 (1.1, 2.4)**	**0.028**		0.9 (0.8, 1.1)	0.329		1.2 (0.9, 1.7)	0.197
Socioeconomic status	Most poor		1.0			1.0			1.0	
	Very poor		0.9 (0.5, 1.4)	0.538		0.9 (0.7, 1.2)	0.478		0.8 (0.5, 1.3)	0.381
	Poor		1.1 (0.7, 1.6)	0.824		0.9 (0.8, 1.1)	0.514		1.0 (0.6, 1.6)	0.906
	Less poor		0.8 (0.5, 1.4)	0.520		**0.7 (0.5, 0.9)**	**0.002**		0.7 (0.4, 1.1)	0.155
	Least poor		1.0 (0.6, 1.8)	0.976		**0.7 (0.5, 0.9)**	**0.006**		0.9 (0.5, 1.5)	0.601
Setting	Rural		1.0			1.0			1.0	
	Urban		0.8 (0.4, 1.6)	0.519		**0.7 (0.5, 0.9)**	**0.018**		1.2 (0.5, 2.6)	0.740
Ecozone	Forest-like		1.0			1.0			1.0	
	Savannah-like		0.6 (0.3, 1.2)	0.151		**1.5 (1.2, 1.9)**	**<0.001**		0.6 (0.3, 1.1)	0.108
Drinking water source	Tap/pump water		1.0			1.0			1.0	
	Well water		**1.7 (1.0, 2.7)**	**0.040**		**1.3 (1.1, 1.7)**	**0.009**		**2.1 (1.3, 3.2)**	**0.001**
	River water		1.1 (0.6, 1.8)	0.739		**1.3 (1.0, 1.7)**	**0.041**		1.5 (0.9, 2.3)	0.090
Sanitation	No latrine		1.0			1.0			1.0	
	Traditional latrine		0.9 (0.5, 1.5)	0.592		0.9 (0.7, 1.1)	0.424		0.9 (0.6, 1.5)	0.806
	Modern latrine		1.1 (0.5, 2.4)	0.867		**0.6 (0.5, 0.8)**	**0.002**		0.5 (0.3, 1.1)	0.092
Hand washing	No regular hand washing		1.0			1.0			1.0	
	Regular hand washing		**0.6 (0.4, 0.8)**	**0.001**		0.9 (0.7, 1.0)	0.078		0.7 (0.5, 1.1)	0.120
Water-related activity	Had no activity		1.0			1.0			1.0	
	Had activity		**2.2 (1.3, 3.6)**	**0.002**		1.0 (0.8, 1.3)	0.832		**2.5 (1.7, 3.6)**	**<0.001**
Deworming	Had no treatment in the past 2 weeks		1.0			1.0			1.0	
	Had treatment in the past 2 weeks		0.8 (0.5, 1.4)	0.471		**0.8 (0.7, 1.0)**	**0.022**		**0.6 (0.4, 1.0)**	**0.025**
Malaria treatment	Had no treatment in the past 2 weeks		1.0			1.0			1.0	
	Had treatment in the past 2 weeks		1.5 (1.0, 2.4)	0.057		0.9 (0.7, 1.0)	0.134		1.0 (0.7, 1.4)	0.963

Parasite mono- and co-infection prevalences and risk factors associated stem from a national survey conducted in Côte d'Ivoire between November 2011 and February 2012. Children from 92 different survey locations were parasitologically tested and socioeconomic status assessed together with their hygiene and health treatment behavior. The prevalences are given in percentages. Associations were assessed using a multinomial regression model adjusted for soil-transmitted helminths and accounting for cluster effects at school unit. Statistically significant (p<0.05) RRRs are highlighted in bold.

RRR = relative risk ratio.

CI = confidence interval.

## Discussion

To our knowledge, this is the first nation-wide survey carried out in Côte d’Ivoire to assess the status and spatial patterns of helminth and *Plasmodium* infection in the school-aged population. Thus far, most parasitological studies in Côte d’Ivoire focused on known high-endemicity areas, such as the region of Man in the western part of the country investigating *S*. *mansoni* [26]–[28] or the Taabo health and demographic surveillance system (HDSS) in south-central Côte d’Ivoire focusing on malaria and helminth infection [29]–[34]. The current survey was conducted over a 4-month period during the dry season in order to minimize seasonal effects in the investigated parasites. *Plasmodium* spp. prevalence was high and quite evenly distributed across the country, whilst *Schistosoma* spp. infection showed a highly focal distribution. Hookworm is the predominant STH species, which corroborates with previous studies [32], [35], with the highest prevalence found in Depingo (70%) in the north-eastern part of the country.

As expected, *P. falciparum* was the predominant malaria parasite throughout Côte d’Ivoire. We found a significant difference in infection prevalence among males and females, but no difference in the two age groups considered (5–9 and 10–16 years). Our observations are consistent with studies from Kenya and Mali that reported significant sex difference of *Plasmodium* infection in childhood [36], [37]. Though studies have shown that children aged below 10 years were more often infected than their older counterparts in stable transmission areas [38], [39], this pattern was not significant in the present study despite the observed higher parasitemia in the younger age group. In general, both the prevalence and parasitemia decrease with age, since people acquire semi-immunity due to sustained exposure to infectious mosquito bites [40], [41].

The prevalence and intensity of helminth infections were assessed through the presence of helminth eggs in stool and the presence of microhematuria in urine. Hookworm infection (17.2%) was most prevalent, followed by *S. haematobium* (proxied by microhematuria; 5.7%), *S. mansoni* (3.7%), *A. lumbricoides* (1.9%), and *T. trichiura* (1.3%). With regard to previous studies in Côte d’Ivoire [29], [42], [43], these helminth infection prevalences are very low. However, one has to consider the fact that for the present study, the school locations were selected using the lattice plus close pairs design, as opposed to preceding studies, which usually focused on specific and known high-risk areas, i.e., villages in close proximity to large man-made lakes and deprived rural settings [44], [45]. Nonetheless, similarities in prevalence with regard to previous studies were found. In the present study the highest prevalence for *S. mansoni* was found in Zoukouboué, in the western part of the country, which is consistent with previous investigations, reporting intestinal schistosomiasis more frequently in the West, where local prevalence rate of 90% or higher have been found [26], [27], [46]–[48]. Meanwhile, *S*. *haematobium* was found to be more prevalent in the South and South-East with the highest prevalence estimated in Banguié (79.6%), which confirms findings from previous studies [44]. The other helminth species varied less in terms of spatial heterogeneity, hence, were present across the country, but with a tendency of higher prevalence in rural compared to urban settings [49].

Our nation-wide parasitological survey revealed that 13.5% and 5.5% of school-aged children had a concurrent STH-*Plasmodium* and *Schistosoma-Plasmodium* infection, respectively, which is somewhat lower than reported before in smaller studies. Righetti and colleagues reported a 27.9% co-infection rate with hookworm and *Plasmodium* among school-aged children in the Taabo HDSS in south-central Côte d’Ivoire [32]. In a single village in western Côte d’Ivoire, Raso and colleagues reported that 75% of community members harbored concomitant infections with *Plasmodium*, helminths, and intestinal protozoa [24]. Helminth-*Plasmodium* co-infections have also been investigated elsewhere in Africa and observed co-infection prevalences ranged from 16% in Uganda and Cameroon, to 26.5% in Tanzania, to 60% in Nigeria [15], [50]–[52]. Given that considerable efforts are currently undertaken to control malaria and helminthiases in Côte d’Ivoire, it is conceivable that the observed co-infection from the national survey might be partly a result of these activities. It must be noted, however, that our survey was carried out only a few months after termination of a prolonged socio-political crisis [17] and national control programs were only just about to restart their activities.

The results from the multinomial logistic regression model identified sex, lack of access to anthelmintic drugs, source of drinking water, and leisure activities in or close to water bodies as determinants common to both STH-*Plasmodium* and *Schistosoma*-*Plasmodium* co-infections. With the exception of sex and contact to water, these patterns might be partly explained by socioeconomic and related factors that were not well captured with our household wealth proxy. In fact, a recent study from south-central Côte d’Ivoire could show that poorer households were less likely to use safe drinking water sources and appropriate sanitation facilities [34]. Furthermore, for both co-infection models, results showed that children were less likely to harbor *Plasmodium* mono-infections if they reported to have received anthelmintic treatment the past 2 weeks and reported to have latrines at home. Though this might as well be related to socioeconomic related factors, such link between *Plasmodium* infections and anthelmintic treatment has been observed in a previous randomized controlled trial from Nigeria among preschool-aged children [53]; following repeated anthelmintic treatment, a reduced increase of the *Plasmodium* infection prevalence in the treatment group compared to the control group coincided with the reduction of *A. lumbricoides* infection prevalence and intensity. It was thus suggested that infection with *A. lumbricoides* hinders the immune system to appropriately respond to clear the *Plasmodium* infection.

Both, the STH-*Plasmodium* and the *Schistosoma*-*Plasmodium* multinomial regression models revealed a higher risk for co-infection related to activities involving close contact to water. Schistosomiasis is a water-borne disease and prolonged contact with cercariae-infested water bodies is a known risk factor [54]. Considering that co-infection is mainly driven by the less frequent species; in this case *Schistosoma* spp., this may explain the finding of an increased risk for mono- and co-infection in the *Schistosoma*-*Plasmodium* model. A potential explanation for the positive association of water contact with STH-*Plasmodium* co-infection could be seen in the general activity patterns of children reporting it. To reach water sites children move in the surroundings of the villages and thus may be more likely to be exposed to open defecation grounds, which represent major transmission sites for hookworm infection, the predominant STH species identified [34].

Ecozone was neither associated with STH-*Plasmodium* nor with *Schistosoma-Plasmodium* co-infection, though STH and *Plasmodium* mono infections showed a relation to ecozone. The fact that no link between co-infections and ecozone was found might suggest that in Côte d’Ivoire, patterns of helminth-*Plasmodium* co-infection are perhaps more influenced by socio-demographic and socio-cultural factors rather than environmental factors. In fact, in a study from East Africa, the pattern of helminth-*Plasmodium* co-infection was governed by the distribution of the less common pathogen [55]. In our study, helminths were far less common than *Plasmodium*, and therefore our results support the aforementioned study. Interestingly, prior research carried out elsewhere in Africa and Asia found a clear link of single and co-infection with the environment [56]–[58]. It will be interesting to monitor helminth-*Plasmodium* co-infection in face of ongoing demographic (e.g., urbanization) and environmental transformation (e.g., deforestation).

Taken together, our results suggest that interactions between parasites exist that can have an impact on control in Côte d’Ivoire and elsewhere in countries where helminths and *Plasmodium* co-exist. We mainly found positive interactions between parasite species; such interactions have been shown to synergistically affect morbidity outcomes [59]. In particular our results showed a positive association between STHs and *Plasmodium*, most importantly with hookworm. Therefore, interventions targeting helminthiases might have beneficial effects beyond the targeted disease, as they might also affect *Plasmodium* infection, which would provide a strong rationale for integrated control approaches that concurrently target STHs and malaria. Clearly, our results from multinomial co-infection models confirmed that interventions against one parasite can positively impact on other infection outcomes, such as in the case of anthelmintic treatment. Combining the distribution of LLINs to the entire population as it is planned by the national malaria control program in Côte d’Ivoire, in conjunction with administration of anthelmintic drugs could thus have high beneficial effects for the population. In addition, such combined interventions have the potential of highest cost-effectiveness, which is of particular relevance in the context of resource scarcity of developing countries.

Our study has several limitations that are offered for consideration. First, given the large scale of the work and constrained financial and human resources, we could only collect single stool, urine, and blood samples for diagnostic work-up. Clearly, more intensive sampling over multiple days and use of a suite of diagnostic methods would have resulted in higher prevalence estimates, and hence higher rates of co-infection [60]–[62]. Second, *S. haematobium* was indirectly assessed using reagent strips and, although the method is a useful screening tool, sensitivity and specificity are imperfect compared to urine filtration considered the ‘gold’ standard. (sensitivity range, 64.8–100%; specificity range, 89.6–93%) [63], [64]. Third, the study was conducted during the dry season. This might lead to underestimate *Plasmodium* spp. prevalence, and hence an underestimated co-infection rate. In fact, the breeding sites of *Anopheles* spp., acting as the vector for *Plasmodium* spp. depend greatly on the availability of stagnant water bodies [58], [65]. Fourth, assessment methods of the malaria parasite used in this study have some shortcomings. Although we employed two methods for the diagnosis of *Plasmodium* spp. (RDT and standard microscopy), it is conceivable that a polymerase chain reaction (PCR) approach would have diagnosed additional cases. The RDT used in our study only identified *P. falciparum*, but this was deemed acceptable, as this is the predominant malaria species throughout Côte d’Ivoire and neighboring countries. At low levels of parasitemia (e.g., 5 parasites/μl of blood), microscopy lacks sensitivity and requires highly trained technicians to accurately diagnose such infections [66], [67].

### Conclusion

Our results confirm that malaria transmission is extremely high across Côte d’Ivoire, despite heightened efforts to reduce the public health burden of this parasitic disease. Malaria is overlapping with helminth infection, and hence, polyparasitism is the norm rather than the exception among children. Co-infection might negatively affect the health and welfare of infected children, including impairment of physical fitness and their ability to attend and successfully complete school with myriad implications for social and work productivity in older age [68]. Hence, our findings should raise awareness and help in designing cost-effective control and mitigation strategies to fight malaria and helminthiases in Côte d’Ivoire and elsewhere in the humid tropics. Further studies need to appraise in greater detail the effect of climatic, environmental, and socioeconomic determinants, as well as how control programs impact on the distribution of single and multiple species parasite infections.

## Supporting Information

Checklist S1 STROBE checklist.
(DOCX)

Table S1 Associations between parasite infections from multivariate logistic regression models accounting for cluster effect at the unit of school.
(DOCX)
