# Supplementary material for: Infection and Co-infection with Helminths and Plasmodium among School Children in Côte d’Ivoire: Results from a National Cross-Sectional Survey
Source: PLoS Negl Trop Dis. 2014 Jun 5;8(6):e2913. doi: 10.1371/journal.pntd.0002913 (PMC4046940; doi:10.1371/journal.pntd.0002913)
Supplement: Table S1 — Associations between parasite infections from multivariate logistic regression models accounting for cluster effect at the unit of school. (DOCX) [file pntd.0002913.s002.docx]

**Table S1.** Associations between parasite infections.

| **Outcome variable** | **Covariate** | **OR (95% CI)** | **P-value** | **Adjusted OR (95% CI)*** | **P-value** |
| --- | --- | --- | --- | --- | --- |
| Soil-transmitted helminths | *S. mansoni* | 1.4 (0.9, 2.2) | 0.088 | ND | ND |
|  | *P. falciparum* | **1.5 (1.3, 1.7)** | **<0.001** | **1.4 (1.2, 1.7)** | **<0.001** |
| Hookworm | *A. lumbricoides* | 1.8 (0.9, 3.7) | 0.106 | 1.7 (0.8, 3.6) | 0.181 |
|  | *T. trichiura* | **3.7 (1.8, 7.6)** | **0.001** | **3.5 (1.6, 7.6)** | **0.002** |
|  | *S. mansoni* | **1.6 (1.0, 2.5)** | **0.039** | ND | ND |
|  | *P. falciparum* | **1.7 (1.4, 2.0)** | **<0.001** | **1.6 (1.3, 1.9)** | **<0.001** |
| *A. lumbricoides* | Hookworm | 1.8 (0.9, 3.6) | 0.121 | 1.7 (0.9, 3.5) | 0.181 |
|  | *T. trichiura* | **11.6 (5.4, 24.8)** | **<0.001** | **11.4 (5.2, 24.9)** | **<0.001** |
|  | *P. falciparum* | 0.7 (0.5, 1.1) | 0.169 | 0.7 (0.5, 1.1) | 0.172 |
| *T. trichiura* | Hookworm | **3.6 (1.8, 7.4)** | **<0.001** | **3.6 (1.8, 7.4)** | **<0.001** |
|  | *A. lumbricoides* | **11.1 (5.3, 22.9)** | **<0.001** | **11.1 (5.3, 22.9)** | **<0.001** |
|  | *P. falciparum* | 0.5 (0.2, 1.1) | 0.099 | 0.5 (0.2, 1.1) | 0.099 |
|  | *P. ovale* | 6.5 (0.7, 59.1) | 0.094 | 6.5 (0.7, 59.1) | 0.094 |
| *S. mansoni* | Hookworm | **1.6 (1.1, 2.4)** | **0.024** | ND | ND |
| *S. haematobium* | *P. falciparum* | 0.7 (0.6, 1.0) | 0.096 | 0.8 (0.6, 1.0) | 0.093 |
| *Plasmodium* spp. | Hookworm | **1.6 (1.4, 1.9)** | **<0.001** | **1.6 (1.3, 1.9)** | **<0.001** |
|  | *T. trichiura* | 0.5 (0.2, 1.3) | 1.131 | 0.5 (0.2, 1.3) | 0.131 |
|  | *S. haematobium* | 0.8 (0.6, 1.0) | 0.073 | 0.8 (0.6, 1.0) | 0.067 |
| *P. falciparum* | Hookworm | **1.7 (1.4, 2.0)** | **<0.001** | **1.6 (1.3, 1.9)** | **<0.001** |
|  | *A lumbricoides* | 0.7 (0.5, 1.2) | 0.194 | 0.7 (0.5, 1.2) | 1.191 |
|  | *T. trichiura* | 0.5 (0.2, 1.1) | 0.096 | 0.5 (0.2, 1.1) | 0.097 |
|  | *S. haematobium* | 0.8 (0.6, 1.1) | 0.114 | 0.8 (0.6, 1.1) | 0.106 |
|  | *P. malariae* | 1.3 (0.9, 1.8) | 0.169 | 1.3 (0.9, 1.8) | 0.185 |
| *P. malariae* | *P. falciparum* | 1.3 (0.9, 1.8) | 0.175 | ND | ND |

Parasite associations stem from a national survey conducted in Côte d'Ivoire between November 2011 and February 2012. Children from 92 different survey locations were parasitologically tested. The parasite-parasite associations were assessed by using multivariate logistic regression models that account for cluster effects at the unit of the school. Statistically significant (p<0.05) odds ratios are highlighted in bold.

OR=odds ratio.

CI=confidence interval.

ND=not determined.

*Adjusted for age and sex groups.
